# A novel large animal model of smoke inhalation-induced acute respiratory distress syndrome

**DOI:** 10.1186/s12931-021-01788-8

**Published:** 2021-07-07

**Authors:** Premila D. Leiphrakpam, Hannah R. Weber, Andrea McCain, Roser Romaguera Matas, Ernesto Martinez Duarte, Keely L. Buesing

**Affiliations:** 1 Department of Surgery, College of Medicine, University of Nebraska Medical Center, Omaha, NE 68198-3280 USA; 2 Department of Mechanical and Materials Engineering, University of Nebraska-Lincoln, Lincoln, NE USA; 3 Department of Pathology and Microbiology, College of Medicine, University of Nebraska Medical Center, Omaha, NE USA

**Keywords:** Smoke inhalation, ARDS, Large animal model, Toxic inhalation injury, Hypoxia, Hypoxemia

## Abstract

**Background:**

Acute respiratory distress syndrome (ARDS) is multifactorial and can result from sepsis, trauma, or pneumonia, amongst other primary pathologies. It is one of the major causes of death in critically ill patients with a reported mortality rate up to 45%. The present study focuses on the development of a large animal model of smoke inhalation-induced ARDS in an effort to provide the scientific community with a reliable, reproducible large animal model of isolated toxic inhalation injury-induced ARDS.

**Methods:**

Animals (n = 21) were exposed to smoke under general anesthesia for 1 to 2 h (median smoke exposure = 0.5 to 1 L of oak wood smoke) after the ultrasound-guided placement of carotid, pulmonary, and femoral artery catheters. Peripheral oxygen saturation (SpO_2_), vital signs, and ventilator parameters were monitored throughout the procedure. Chest x-ray, carotid, femoral and pulmonary artery blood samples were collected before, during, and after smoke exposure. Animals were euthanized and lung tissue collected for analysis 48 h after smoke inhalation.

**Results:**

Animals developed ARDS 48 h after smoke inhalation as reflected by a decrease in SpO_2_ by approximately 31%, PaO_2_/FiO_2_ ratio by approximately 208 (50%), and development of bilateral, diffuse infiltrates on chest x-ray. Study animals also demonstrated a significant increase in IL-6 level, lung tissue injury score and wet/dry ratio, as well as changes in other arterial blood gas (ABG) parameters.

**Conclusions:**

This study reports, for the first time, a novel large animal model of isolated smoke inhalation-induced ARDS without confounding variables such as cutaneous burn injury. Use of this unique model may be of benefit in studying the pathophysiology of inhalation injury or for development of novel therapeutics.

## Introduction

Acute respiratory distress syndrome (ARDS) is a serious pulmonary condition in critically ill patients with a reported mortality rate ranging from 30–45% [1, 2]. There has been no significant change in the mortality rate since 1994 [2]. ARDS can be caused by a variety of direct and indirect injuries to the lung, including sepsis, trauma, pneumonia and smoke inhalation/burn injury [3–5]. Understanding the pathophysiological and molecular mechanisms of ARDS is critical for the development of novel therapeutic strategies for ARDS.

ARDS was coined by Ashbaugh et al*.* in 1967 to describe an acute onset of tachypnea, hypoxemia, and loss of compliance after a variety of insults [6]. The most current consensus definition for ARDS in clinical setting was published in 2012 as the Berlin criteria [7]. The Berlin criteria base categorization of ARDS on level of hypoxemia measured by the PaO_2_/FiO_2_ (arterial oxygen partial pressure/fraction of inspired oxygen) ratio, positive end-expiratory pressure (PEEP) level, development of bilateral pulmonary infiltrates on chest x-ray, and normal pulmonary capillary wedge pressure (PCWP) within a week of a known clinical insult [7]. The lung injury in ARDS has been reported to undergo three pathophysiological phases: the exudative phase involves damage to the alveolar epithelium leading to increased lung permeability; the proliferative phase involves type II cell proliferation with epithelial cell regeneration, fibroblastic reaction, and remodeling; and the irreversible fibrotic phase, which includes collagen deposition in the lung [8–10]. To understand the development of ARDS, reliable animal models that can mimic these pathophysiological phases are critical. Previous studies have used mouse models for assessment of pulmonary gas exchange and respiratory physiology following controlled induction of ARDS [11, 12]. However, these mouse models have limitations in the induction of mechanical ventilation and collection of blood samples, and are therefore, not amenable for prolonged study essential to mimic the clinical presentations of ARDS. Large animal models have been reported to show better translational potential in the study of ARDS. Swine models are considered an excellent model for pulmonary pathology due to the similarities with humans in terms of anatomy, genetics and physiology [13]. Various studies have used swine models to study lung development [14, 15], acute lung injury (ALI)/ARDS [16, 17] and other diseases.

Smoke inhalation is one of the major causes for the development of ARDS after burn injury, with an approximate 30–90% mortality rate [18, 19]. Several large animal models are available for smoke/burn injury-induced ARDS [20–22]. However, at present, there is no suitable large animal model available to study isolated smoke inhalation-induced ARDS without confounding variables such as cutaneous burn injury.

The rate of ARDS after smoke inhalation injury varies from patient to patient and is dependent upon several variables such as particulate matter concentration, carbon monoxide concentration, volatile compounds in the smoke, etc. Smoke inhalation injury is the primary injury leading to acute lung injury/ARDS in humans involved in house fires; the presence/absence of cutaneous burn injury plays an additive role in the patient’s overall pathophysiology which significantly complicates the medical and surgical management of this patient population. Exposure to indoor air pollution from biomass combustion—as seen during combustion of biomass fuels for cooking and heating used by approximately half of all people in developing countries—is a major source of morbidity and mortality worldwide and continues to be an area of interest for research. As well, the detrimental effect of prolonged exposure to acutely elevated levels of environmental smoke from wildfires or house fires has long-standing interest, especially when studying workplace hazards of firefighters. The main goal of our work presented in the current manuscript was to study isolated smoke inhalation injury without confounding variables such as cutaneous burn, in an attempt to add to current published literature in a way that would be translatable to many real-life situations.

We have developed a large animal, isolated smoke inhalation model to use in our investigations of molecular modification after smoke inhalation and novel therapeutic agents. In the study, pigs were exposed to smoke directly through an endotracheal route to induce lung injury in a controlled environment. Invasive and non-invasive parameters including vital signs, arterial blood gas analysis, and chest x-rays were monitored to pinpoint the development of significant ARDS. IL-6 and histological analysis were performed to understand the pathophysiological profile critical for the development of ARDS in these animals. To our knowledge, this is the first study to develop and detail a large animal model of isolated smoke inhalation-induced ARDS.

## Methods

### Subjects

All the experiments involving animals were approved by University of Nebraska Lincoln (UNL) Institutional Animal Care and Use Committee (IACUC) (protocol # 1674). Female pigs (~ 50 kg, n = 21) were housed in pens and cared for according to USDA (United States Department of Agriculture) guidelines. To our knowledge, female pigs do not have higher susceptibility to ARDS compared to male pigs. Our research collaborators had previously developed a porcine model of severe ARDS from intratracheal lipopolysaccharide using female pigs only, and our use of female pigs in the current study was, in part, to assess reliability and reproducibility in their model using a contrasting lung injury.

Animals were acclimated to the facility for 4–7 days and received food reward training to ease handling and blood draws. Six animals died during the experiments due to either anesthesia/surgical complications or smoke inhalation related complication. We continued our next phase of study (involving efficacy of a novel therapeutic for hypoxia) in some of these animals to reduce cost and number of animals used.

### Smoke delivery system

**Fig. 1:**
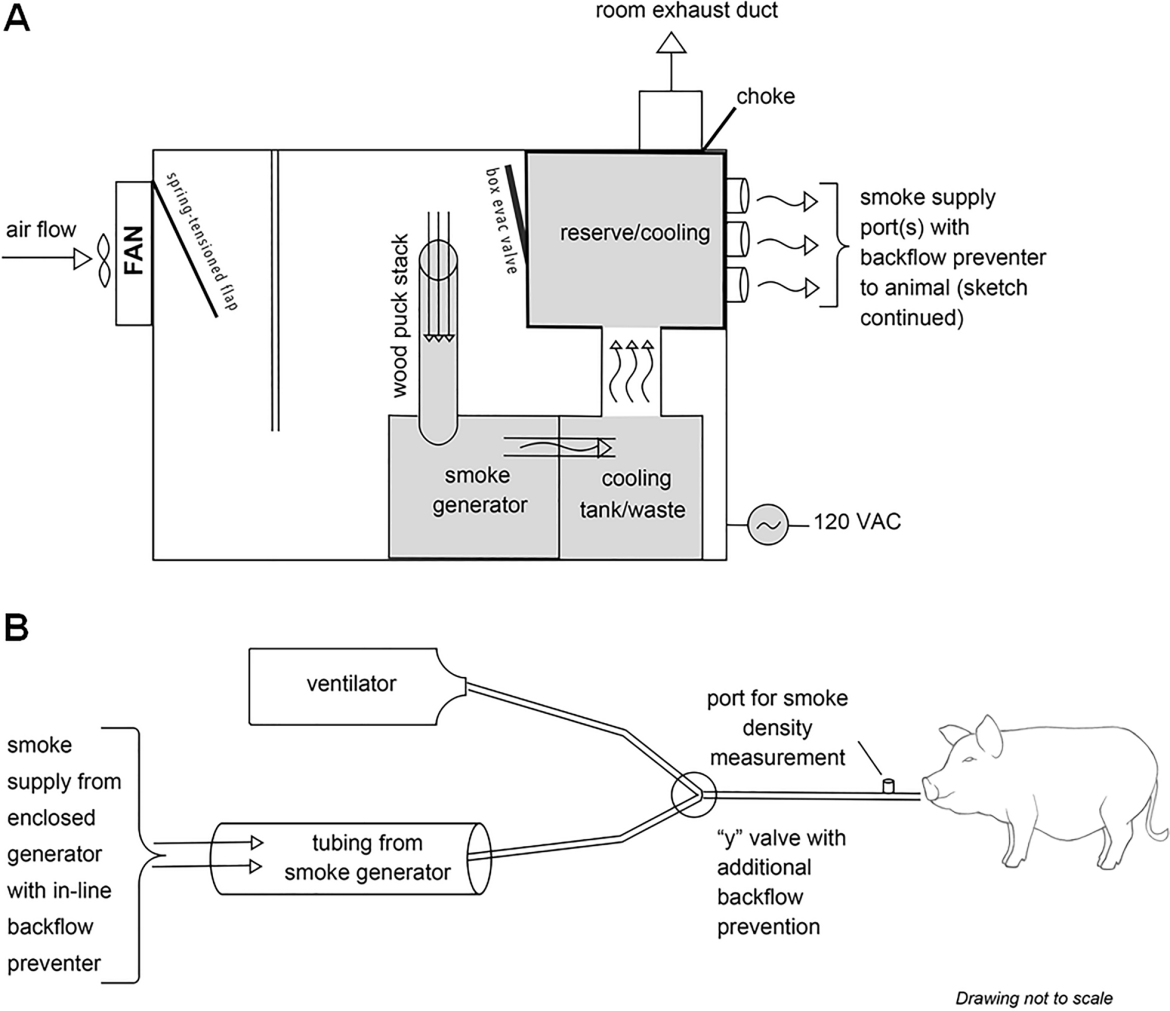
Schematic diagram of the smoke generator system and delivery circuit

Upon completion of all surgical procedures, animals were exposed to oak wood smoke from a custom-made smoke generator connected in parallel to the endotracheal tube. Schematic of the smoke delivery system is shown in Fig. 1.

### Surgical procedures

Sedation for peripheral intravenous catheter (PIV) placement and endotracheal intubation was achieved with a mixture of telazol (4.4 mg/kg), ketamine (2.2 mg/kg) and xylazine (2.2 mg/kg) delivered via intramuscular injection. To assist with intubation, an intravenous bolus dose of fentanyl (0.05 mg/kg) and/or propofol (2–4.4 mg/kg) was given as needed. Baseline chest x-rays were obtained (portable x-ray unit EPX-F2800, Ecotron Co. Ltc; wireless digital flat panel detector Mars1417V-TSI, iRay Technology, Shanghai, China) prior to smoke inhalation, and at 24 and 48 h after smoke inhalation. An endotracheal tube (#7–8 cuffed; MWI Animal Health, Boise, ID, USA) was inserted into the trachea and animals were ventilated at a tidal volume (TV) of 6 mL/kg and PEEP of 5 cmH_2_O (Newport HT70, Medtronic, Minneapolis, MN). Respiratory rate (RR) was adjusted to maintain eucapnia as monitored by end-tidal CO_2_ (ETCO_2_). The fraction of inspired oxygen (FiO_2_) was set at 50% during surgical procedures (central venous & arterial catheter placement), then titrated down to 21% and maintained throughout the experiment. 50% FiO_2_ was used to reduce stress to animal during surgical procedures and lasted for 30–40 min. Non-invasive monitoring included blood pressure taken by cuff placed around the animal's hind leg, peripheral oxygen saturation (SpO_2_), heart rate (HR) and ETCO_2_ recorded via the Surgivet monitor (Smiths Medical, Dublin, OH). Continuous IV sedation containing midazolam (0.4–0.7 mg/kg/h), fentanyl (0.03–0.1 mg/kg/h) and propofol (0.2–0.4 mg/kg/min) and maintenance IV fluids (10 mL/kg/h normal saline) were given throughout the procedure via a quadruple-lumen central venous catheter (8.5Fr × 16 cm, Arrow International) placed in the internal jugular vein. Core temperature was monitored by rectal probe and a circulating warming blanket was used to prevent body cooling. A urinary catheter was placed to monitor output.

Using sterile technique and ultrasound guidance (Butterfly iQ, Butterfly Network, New York City, NY), carotid artery (CA) and femoral artery (FA) access catheters were placed for serial lab draws and invasive blood pressure monitoring (18 GA 16 cm; Femoral Arterial Line Catherization Kit; Teleflex, Morrisville, NC). Pulmonary artery (PA) catheter (8F × 110 cm Swan-Ganz CCOmbo Thermodilution Catheter; Edwards Lifesciences, Irvine, CA) was placed in the internal jugular vein under ultrasound guidance. The CA and PA access ports were connected to Surgivet monitor and Vigilance II monitor, respectively (Edwards Lifesciences, Irvine, CA) with transducers (Meritans DTXPlus, Disposable Pressure Transducer with EasyVent; Merit Medical, South Jordan, UT, USA). Invasive arterial blood pressure, central venous pressure (CVP), pulmonary artery pressure (PAP), cardiac output (CO), mixed venous oxygen saturation (SmvO_2_), and central (core) temperature were monitored throughout the study. Blood samples were drawn from the CA, FA and PA catheters for the measurement of baseline blood gas prior to smoke inhalation and at pre-determined time intervals throughout the study period (ABL80 FLEX CO-OX, Radiometer, Brea, CA). To maintain patency, catheters were flushed throughout the experiment with 3–5 mL of sterile saline, and a heparin solution (1:500 dilution in 50% dextrose solution) was infused to fill the volume of the port chosen as a "lock" solution. Sedated/anesthetized animals from survival surgeries were continuously monitored until sternal recumbence was regained. All catheters were removed after smoke inhalation was completed. The surgical procedures were repeated at 48 h after smoke inhalation.

### Smoke inhalation

Upon completion of surgical procedures, animals in the SI group were exposed to oak wood smoke from the custom-made smoke chamber (Fig. 1) through the endotracheal tube. The duration of the smoke exposure was 1 to 2 h starting from 0 h time point. Smoke was generated at room temperature (74–76 deg F) in a controlled manner. Smoke density/particle load was not measured in the current study. The volume of smoke inhaled was approximately 500 L per hour. Invasive and noninvasive parameters were monitored continuously during the experiment. Following smoke exposure, blood samples were collected from arterial ports and PAC. Animal was continuously monitored until recovery from general anesthesia. Smoke exposure was stopped immediately if the animal developed hemodynamic instability, which was determined by hypotension (systolic blood pressure less than 60) and irreversible desaturation (SpO_2_ less than 70% despite rescue maneuvers such as increase in inspired percentage of oxygen).

### Ventilator parameters

On the day of the smoke inhalation, the ventilator parameters were maintained at values: tidal volume, 6 mL/kg = 270-360L/min; respiratory rate, 18–30/min; PEEP 5 mmHg; and FiO_2_, 21–34% (Table 1). During the 48 h after smoke inhalation injury, animals were extubated and maintained on room air.

**Table 1:** Ventilator parameters

Parameters	Baseline	SI 2 h	Post SI 24 h	Post SI 48 h
TV (mL/kg)	270–360	270–360	–	220–280
RR (bpm)	18–30	18–30	–	16–24
FiO_2_ (%)	21–34	21–34	21	21
PEEP (cmH_2_O)	5	5	–	0

SI, Smoke inhalation; TV, tidal volume; RR, respiratory rate; bpm, beats per minute; FiO_**2**_, fraction of inspired oxygen; PEEP, positive end expiratory pressure

n = 13

At 48 h after smoke inhalation injury, animals were again placed on the ventilator, surgical procedures of catheter placement were repeated, serial lab, imaging, and BAL sampling was completed, and animals were humanely euthanized per IACUC protocols. On the final study day, ventilator parameters were similar to the day of smoke inhalation with the exception of maintaining PEEP at 0. To reduce the effect of elevated mechanical power applied to the lung, resulting in worsening of acute lung injury through development of ventilator-induced lung injury, we elected to avoid this potential confounder by maintaining the PEEP at zero if drive pressure was noted to be adequate and at safe levels. As noted in Swindle’s authoritative reference (Swindle MM. Swine in the Laboratory: Surgery, Anesthesia, Imaging, and Experimental Techniques. 2007, Second Edition, CRC Press), swine have very fragile pulmonary tissue that can be damaged by hyperinflation, and it is recommended to maintain pressure between 11–20 cm H20. In our animals, drive pressure was maintained in this range without use of PEEP at 48 h post smoke inhalation injury.

### Post-surgical animal monitoring and care

After recovery from surgical anesthesia, all animals were transferred to the post-surgery recovery pen and were monitored 24 h/day by trained personnel. Chest x-rays were taken and blood samples drawn for ABG analysis at 24 h and 48 h following final smoke exposure. Animals (n = 7) were humanely euthanized at 48 h post SI and a full necropsy was performed; the remainder of animals were utilized to continue our lab’s therapeutic investigations.

### Plasma sample extraction

Blood samples were collected from the CA catheter at baseline, 2 h, and 48 h time points in lithium heparin BD Microtainer tubes (Becton, Dickinson and Company, Franklin Lakes, NJ). Tubes were immediately inverted 8–10 times to assure anticoagulation and centrifuged at 4000*g* for 4 min. Supernatants were collected as plasma samples and stored at − 80 °C until analysis. IL-6 immune assay was performed in samples of 10 animals using IL-6 Quantikine® ELISA kit, catalog No#P6000B (R&D Systems, Inc., Minneapolis, MN) on plasma samples following manufacturer’s protocol.

### Bronchoalveolar lavage (BAL)

BAL of pig lungs was performed at baseline, 2 h, and 48 h time points using a bronchoscope in a set of 6 intubated animals. 10 ml of sterile normal saline was instilled to the secondary and tertiary bronchi through the bronchoscope and ~ 5 ml of the fluid was collected for analysis. BAL fluid samples were centrifuged immediately at 400*g* at 4 °C for 10 min and supernatants were at stored at − 80 °C until analysis. Total protein quantification was performed in samples using Pierce™ BCA (Bicinchoninic Acid) Protein Assay Kit (Thermo Fisher Scientific Inc. Waltham, MA) following manufacturer’s protocol. IL-6 immune assay was performed using IL-6 Quantikine® ELISA (Enzyme-linked immunosorbent assay) kit, catalog No#P6000B (R&D Systems, Inc., Minneapolis, MN) on BAL fluid samples following manufacturer’s protocol.

### Tissue collection

Necropsy was performed in 7 animals (control, n = 2; SI, n = 5). At necropsy, lung tissues were collected from all five lobes; upper, middle and lower lobes of right lung and upper and lower lobes of left lung for histological examination and pulmonary edema assessment. Tissues for histology were immediately placed in 10% neutral buffer formalin fixative for approximately 24 h. Formalin fixed tissues were placed into 70% ethanol and transferred to the University of Nebraska Medical Center (UNMC) Tissue Science Facility (TSF) for further tissue processing and embedment in paraffin blocks.

### Lung injury score

The lung tissue of all five lobes in 10% neutral formalin was dehydrated in graded concentrations of ethanol solution and cleared in xylene. The tissue samples were then paraffin-embedded, sectioned with 4-μm thickness, and stained with hematoxylin and eosin at the UNMC Tissue Sciences Facility using automated Ventana Discovery Ultra (Roche Diagnostics, Indianapolis, IN) as per manufacturer’s protocol. For each animal staining was performed in five sections corresponding to the five lobes of lung. An independent pathologist performed a blinded examination of the tissues under light microscope. Ten fields of each lung tissue section were examined at magnification X400. The severity of the lung injury was scored by the criteria of alveolar edema, intra-alveolar hemorrhage, and leukocyte infiltration. Alveolar edema and intra-alveolar hemorrhage were scored on a scale from 0 to 3; where 0 ≤ 5% of maximum pathology, 1 = mild (< 10%), 2 = moderate (10–20%), and 3 = severe (20–30%). Leukocyte infiltration was also scored on a scale from 0 to 3; where 0 = absence of extravascular leukocytes, 1 ≤ 10, 2 = 10–45, and 3 ≥ 45 leukocytes.

### Wet-to-dry weight (W/D) ratio

Lung tissues (n = 6) were dried in an incubator at 60 °C for 5 days and weighed again (dry weight). The W/D ratio was calculated as the ratio of the wet weight to the final dry weight as described elsewhere [23].

### Ki67 immunohistochemistry

Immunostaining for Ki67 was performed on pig SI and control formalin‐fixed, paraffin‐embedded lung tissues sections using 1:200 concentration of Ki67 antibody (#ab16667, Abcam Inc, Cambridge, MA) at UNMC Tissue Sciences Facility using automated Ventana Discovery Ultra (Roche Diagnostics, Indianapolis, IN) in lung tissue samples of 5 animals (control, n = 2; SI, n = 3) as per manufacturer’s protocol. For each animal staining was performed in five tissue sections corresponding to the five lobes of lung. Specimens were processed on the same day to eliminate any variability in conditions. An independent pathologist performed a double-blinded examination of the tissue slides under a light microscope. A total of 2000 cells were counted at magnification of X400 and the percentage of Ki67 positive cells were calculated.

### Lung tissue lysate preparation

Fresh frozen lung lobe tissues with highest injury score (n = 5) were homogenized using VWR® Mini Bead Mill Homogenizer (VWR International LLC., Radnor, PA) following manufacturer’s protocol. Briefly, frozen tissues of two control and three SI animals were washed in cold X1 PBS, and 30 mg of each tissue was placed separately in a 2 mL tube containing 2.8 mm ceramic beads and 750 μl of lysis buffer containing RIPA buffer (Thermo Fisher Scientific Inc. Waltham, MA) and protease inhibitor cocktail (Sigma Aldrich Inc., St. Louis, MO) at room temperature. The samples were homogenized at speed 4 for 60 s. This was followed by incubation in ice for 30 min and centrifugation at 13,000 rpm for 20 min at 4 °C. Protein concentration was determined using Pierce™ BCA (Bicinchoninic Acid) Protein Assay Kit (Thermo Fisher Scientific Inc. Waltham, MA) following manufacturer’s protocol.

### Immunoblot analysis

Protein (50 μg) was separated by SDS‐ polyacrylamide gel electrophoresis and transferred onto PVDF (polyvinylidene fluoride) membrane (Bio-Rad Lab Inc., Hercules, CA) by electro blotting. The membrane was blocked with 5% nonfat dry milk in X1 TBST (50 mM Tris, pH 7.5, 150 mM NaCl, 0.01% Tween 20) for 1 h at room temperature. The membrane was then incubated in primary antibody, IL-6 antibody (#ab6672, Abcam Inc, Cambridge, MA) or β-actin (#4970, Cell Signaling Technology Inc., Danvers, MA) at 1:1000 dilution in X1 TBST with 5% bovine serum albumin (Sigma Aldrich Inc., St. Louis, MO) overnight at 4 °C. The membrane was washed three times with X1 TBST for 10 min each and incubated with HRP‐conjugated secondary antibody (#7074, Cell Signaling Technology Inc., Danvers, MA) at 1:5000 dilution in X1 TBST with 5% nonfat dry milk for 1 h at room temperature. Following washes in X1 TBST, proteins were detected using the enhanced chemiluminescence system (Bio-Rad Lab Inc, Hercules, CA).

### Statistical analysis

Statistical analysis was performed using GraphPad prism 8. One-way ANOVA with Tukey’s post-hoc analysis and paired t test were utilized to generate adjusted “p” values. P-values < 0.05 were considered statistically significant.

## Results

**Fig. 2:**
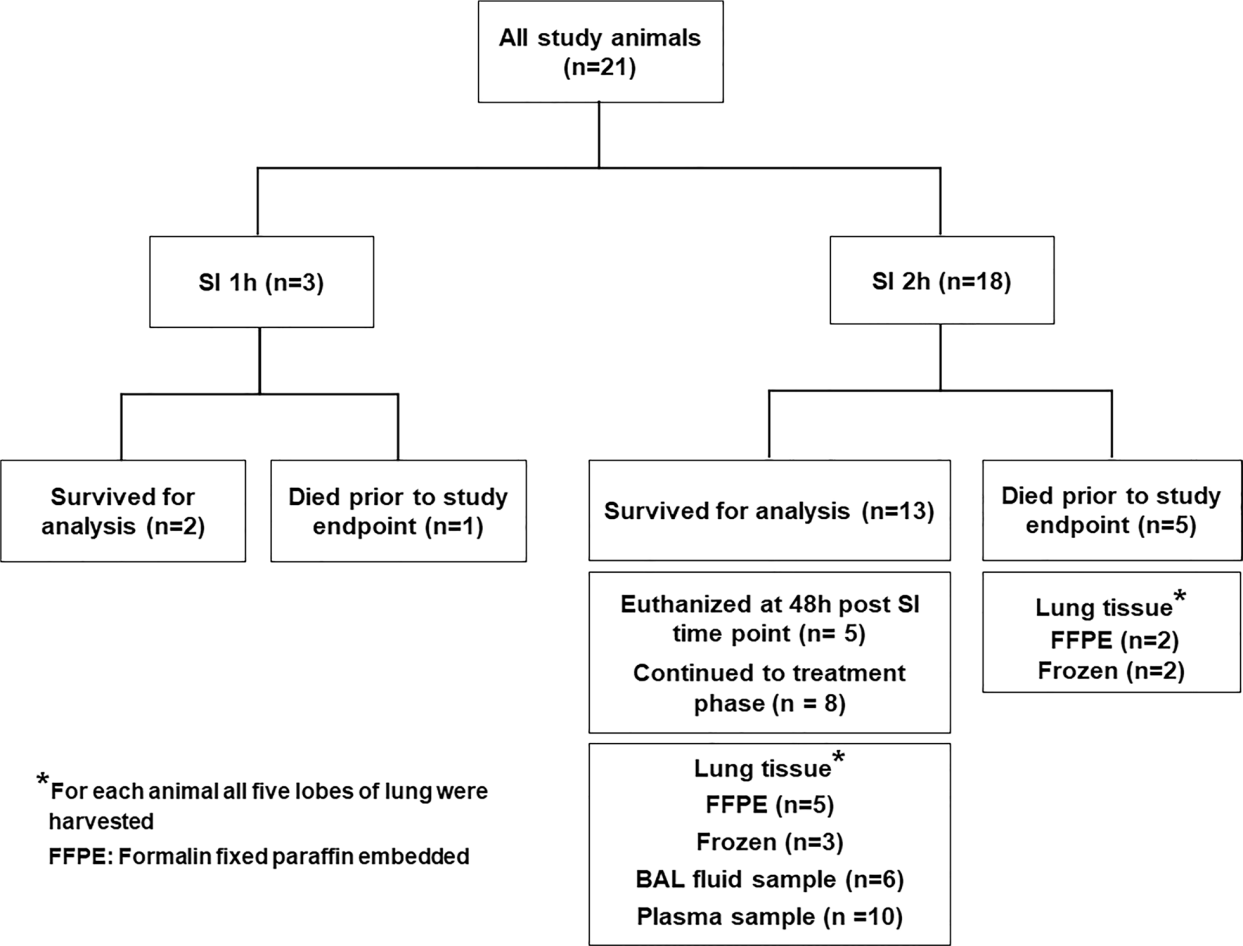
Flow diagram representing animals assigned to different study groups

The study was divided in two phases. The first phase of the study was performed to determine the optimal duration of smoke exposure required for the development of ARDS. Animals were divided according to smoke inhalation time as shown in the flow diagram in Fig. 2. Smoke inhalation for 1 h was designated as “SI 1 h” (n = 3) and smoke inhalation for 2 h was designated as “SI 2 h” (n = 18). Fifteen animals successfully completed smoke inhalation (SI) experiment and survived 48 h post smoke exposure, and six animals died during procedures due to either anesthesia/surgical complications or smoke inhalation-related complication.

### Smoke inhalation reduced peripheral oxygenation in large animal model

**Fig. 3:**
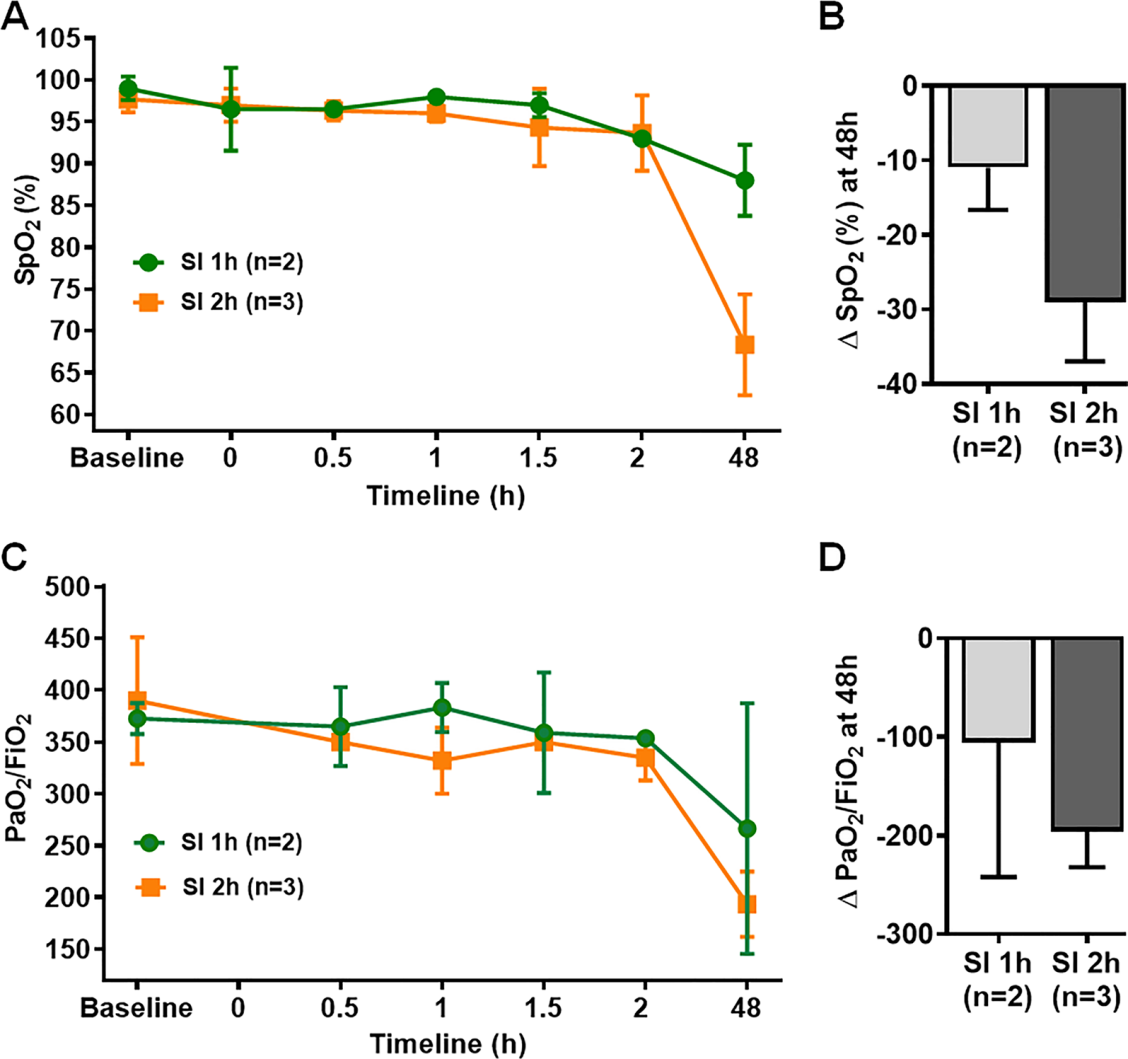
Effect of smoke duration in large animal. **A**, **B** Peripheral oxygen saturation (SpO_2_) level was measured in 1 h- and 2 h-smoke inhalation groups designated as “SI 1 h” and “SI 2 h”, respectively, at different time points from 0 to 48 h. **A** Statistical analysis of the delta SpO_2_ (designated as ΔSpO_2_) between SI 1 h and SI 2 h groups (**B**). ΔSpO_2_ for each group represented the difference in SpO_2_ level between baseline and 48 h post smoke exposure. **C**, **D** The ratio of partial pressure of arterial oxygen (PaO_2_) and fraction of inspired oxygen (FiO_2_), designated as “PaO_2_/ FiO_2_” was measured in SI 1 h and SI 2 h group of animals (**C**). Statistical analysis of the delta PaO_2_/FiO_2_ (designated as ΔPaO_2_/FiO_2_) between SI 1 h and SI 2 h groups (**D**). ΔPaO_2_/FiO_2_ for each group represented the difference in PaO_2_/FiO_2_ level between baseline and 48 h post smoke exposure. Smoke inhalation started at 0 h time point A p value of < 0.05 was considered statistically significant

The SpO_2_ level was measured continuously on the day of the smoke inhalation experiment and at 24 h- and 48 h- post smoke inhalation. The SpO_2_ level following 1 h- or 2 h- smoke inhalation was approximately 95% and started to decrease at 24 h post smoke inhalation in both groups. However, SpO_2_ level significantly dropped in the SI 2 h group at 48 h post smoke exposure (68 ± 6%) compared to baseline (98 ± 2%) and the SI 1 h group (88 ± 4%) (Fig. 3A). The decrease was approximately 30% as reflected by the delta SpO_2_ (ΔSpO_2_) value in SI 2 h group compared 10% in the SI 1 h group (Fig. 3B). In addition, at 48 h post smoke exposure PaO_2_/FiO_2_ ratio was reduced approximately to 193.4 in the SI 2 h group compared to baseline and SI 1 h group values (267–390) (Figs. 3C, D). These results demonstrated that at 48 h post smoke exposure, animals with smoke inhalation for 2 h showed sign of respiratory distress as reflected by decrease in SpO_2_ value below 68% and indicated that there is injury to the lung.

**Fig. 4:**
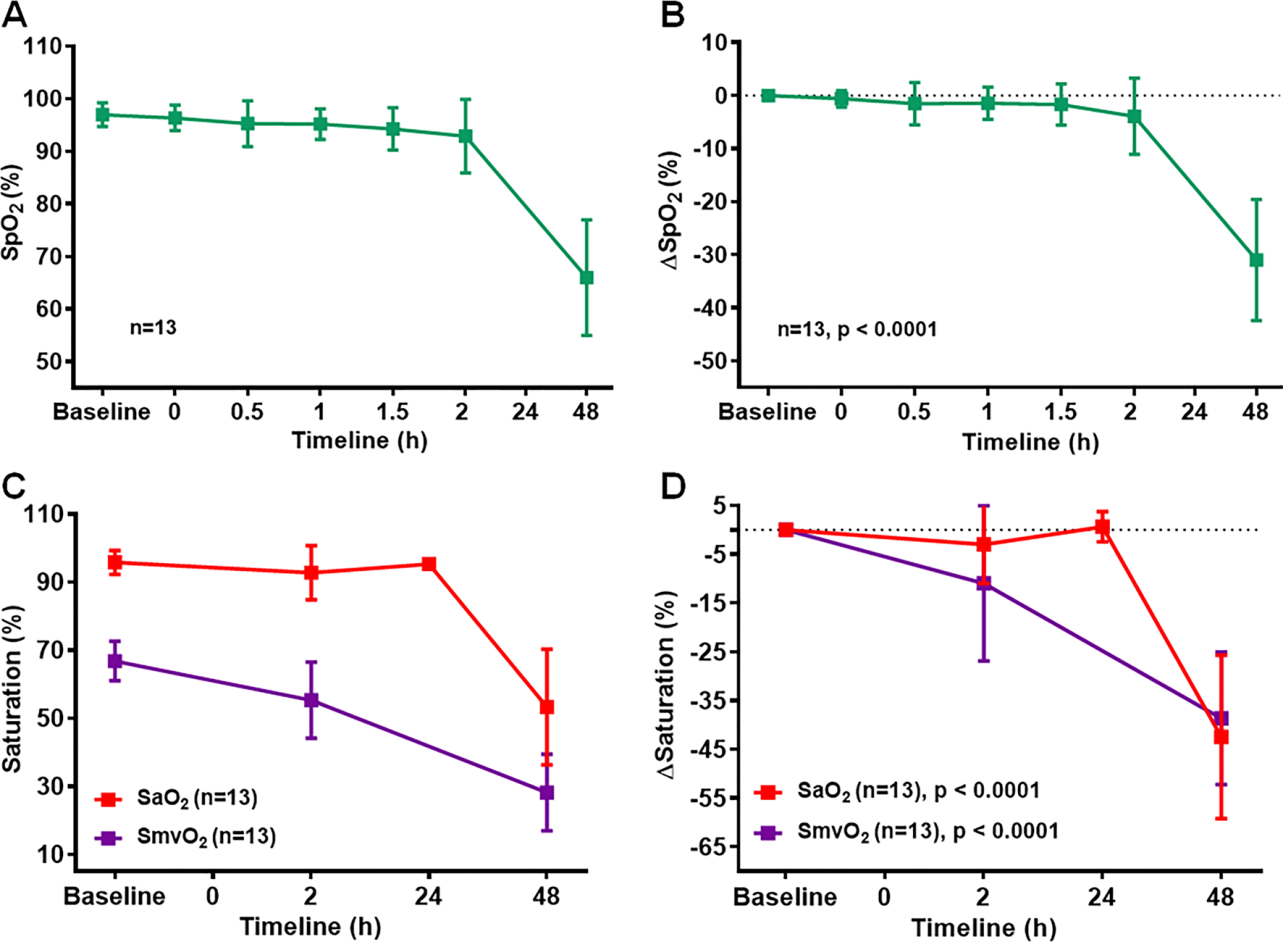
Smoke inhalation reduced oxygen saturation in large animals. **A**, **B** Peripheral oxygen saturation (SpO_2_) level was measured in a different set of 2 h smoke inhalation group designated as “SI” at different time points from 0 to 48 h. **A** ΔSpO_2_ values between baseline and different time points (**B**). **C**, **D** Arterial oxygen saturation (SaO_2_) and mixed venous oxygen saturation (SmvO_2_) levels were measured in SI group at baseline, 2 h, 24 h, and 48 h time points (**C**). Delta SaO_2_ (ΔSaO_2_) and delta SmvO_2_ (ΔSmvO_2_) values were calculated between baseline and different time points (**D**). Delta value for each parameter represented the difference with corresponding baseline values. SaO_2_ level was measured at 24 h for n = 8, SpO_2_ and SmvO_2_ levels were not measured at 24 h time point. Smoke inhalation started at 0 h time point. A p value of < 0.05 was considered statistically significant

For the rest of the study, we concentrated at the 2 h time point for duration of smoke exposure, and repeated experiments to ensure reproducibility of this model. Animals were exposed to smoke for 2 h duration and designated as “SI” animals. Consistent with the result in Fig. 3, we observed a 22–40% decrease in SpO_2_ compared to baseline values (Figs. 4A, B, p < 0.0001). In addition, arterial and mixed venous oxygen saturation (SaO_2_ and SmvO_2_) were reduced by approximately 39–43% compared to the corresponding baseline values (Figs. 4C, D, p < 0.0001).

All results presented to follow are after 2 h smoke exposure.

### Smoke inhalation induced hypoxemia in large animal model

**Fig. 5:**
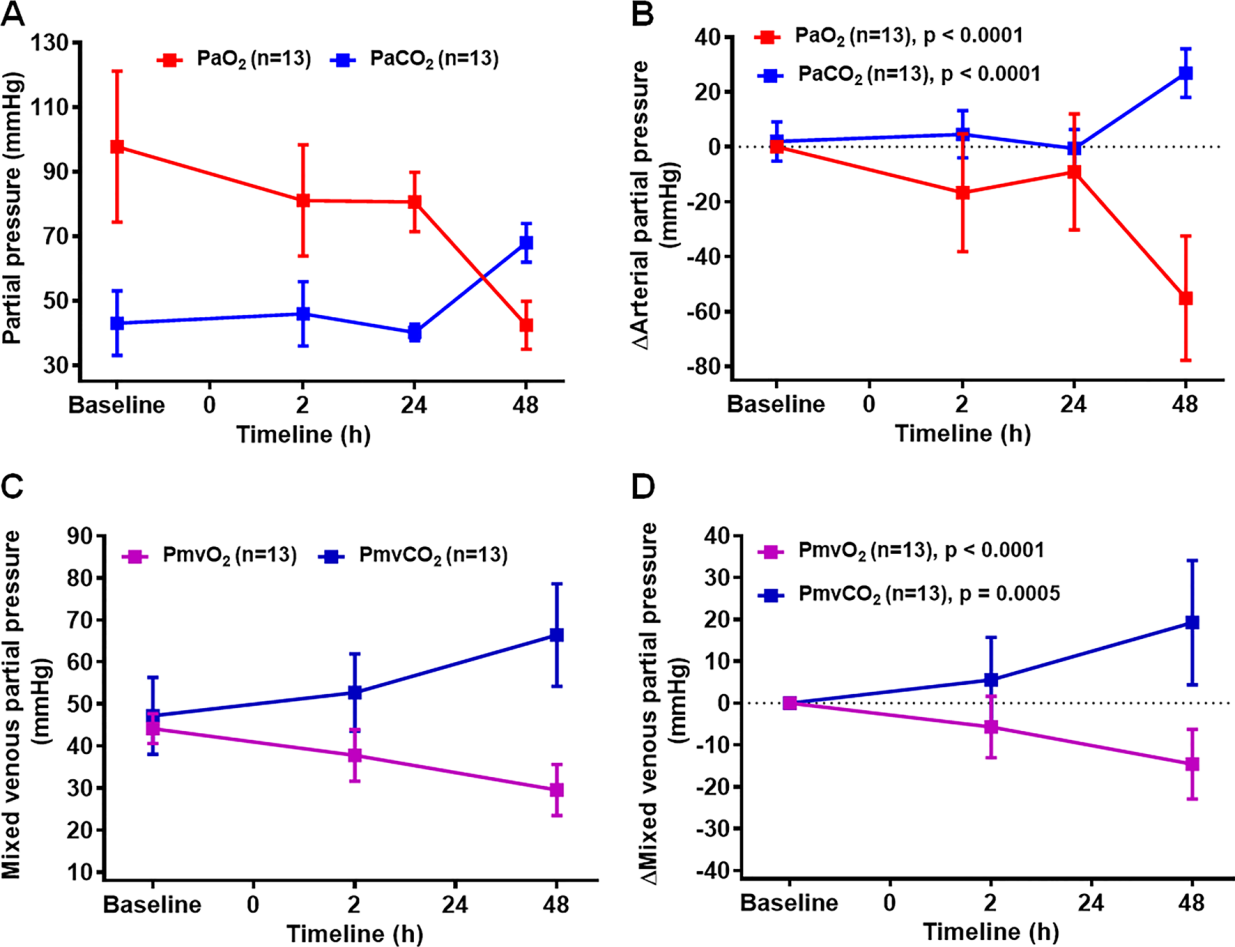
Smoke inhalation reduces PO_2_ with reciprocal increase in PCO_2._ Arterial partial pressure of oxygen (PaO_2_) and carbon-dioxide (PaCO_2_) levels were measured in SI group at baseline, 2 h, 24 h, and 48 h time points (**A**). Delta PaO_2_ (ΔPaO_2_) and delta PaCO_2_ (ΔPaCO_2_) values between baseline and different time points (**B**). Mixed venous partial pressure of pressure of oxygen (PmvO_2_) and carbon dioxide (PmvCO_2_) level were measured in SI group at baseline, 2 h, 24 h, and 48 h time points (**C**). Delta PmvO_2_ (ΔPmvO_2_) and delta PmvCO_2_ (ΔPmvCO_2_) values were calculated between baseline and different time points (**D**). Delta value for each parameter represented the difference with corresponding baseline values. PaO_2_ and PaCO_2_ levels were measured at 24 h for n = 8; PmvO_2_ and PmvCO_2_ levels were not measured at 24 h time line. Smoke inhalation started at 0 h time point. A p value of < 0.05 was considered statistically significant

Arterial blood gas (ABG) was measured from both arterial and mixed venous blood samples throughout the study. The arterial partial pressure of oxygen (PaO_2_) level started to decrease 1 h after smoke exposure and dropped significantly at 48 h post smoke exposure to 43 mmHg from baseline value of 95 mmHg (Fig. 5A). Delta PaO_2_ (ΔPaO_2_) value in Fig. 5B showed a difference of 53 ± 5.8 mmHg (p < 0.0001). As expected, there was a rise in partial pressure of carbon dioxide (PaCO_2_) level (Fig. 5A) with an increase in ΔPaCO_2_ value by 27.5 ± 2.34 mmHg compared to the baseline value (Fig. 5B, p < 0.0001). In correlation with findings from arterial samples, at 48 h post smoke exposure we observed significant reduction in the partial pressure of mixed venous oxygen (ΔPmvO_2_) level (15.12 ± 2.12 mmHg) and corresponding increase in partial pressure of mixed venous carbon dioxide (ΔPmvCO_2_) level (19.23 ± 4.01 mmHg) compared to the baseline values (Figs. 5C, D, p < 0.0001 to = 0.0005). The pH level was also significantly decreased at 48 h post smoke inhalation (Table 2, p < 0.001) with no significant change in the HCO_3_ levels (Table 2). Consistent with the rise in PaCO_2_ value post smoke inhalation, end tidal CO_2_ (ETCO_2_) – which measures the concentration of CO_2_ exhaled at the endotracheal tube—significantly increased from the baseline value of 33.9 ± 12.8 mmHg to 52.63 ± 13.5 mmHg at 48 h post smoke inhalation (Table 2, p = 0.009).

**Table 2:** Hemodynamic parameters

Parameters	Baseline	SI 2 h	Post SI 24 h^a^	Post SI 48 h
Art pH	7.47 ± 0.07	7.45 ± 0.07	7.47 ± 0.04	7.30 ± 0.10***
Art HCO_3_ (mmol/L)	29.33 ± 2.19	30.42 ± 1.80	27.49 ± 3.02	30.09 ± 3.70
ETCO_2_ (mmHg)	33.90 ± 12.8	40.56 ± 11.8	–	52.63 ± 13.5**
Hct (%)	34.35 ± 2.82	34.02 ± 3.46	31.75 ± 2.99	29.58 ± 6.04*
Hb (g/dl)	11.08 ± 0.84	11.04 ± 1.15	10.37 ± 0.94	9.50 ± 1.89*
FO_2_Hb (%)	95.35 ± 3.80	91.74 ± 7.89	–	52.13 ± 16.4***
HR (bpm)	87.17 ± 18.3	92.91 ± 41.63	–	88.75 ± 12.88
Temp (^0^C)	37.18 ± 0.87	36.67 ± 4.43	–	41.27 ± 11.1
MAP (mmHg)	66.50 ± 26.8	72.68 ± 12.8	–	74.83 ± 19.0
PAP (mmHg)	15.25 ± 9.20	14.71 ± 10.2	–	23.84 ± 17.2

Values are calculated as mean ± SD, n = 13. Art pH, arterial pH; Art HCO_3_, arterial bicarbonate; ETCO_2_, end tidal carbon dioxide; Hct, hematocrit; Hb, hemoglobin; FO_2_Hb, fractional oxyhemoglobin MAP, mean arterial pressure; PAP, pulmonary arterial pressure; HR, heart rate; Temp, temperature

** p < 0.01, *** p < 0.001, **** p < 0.0001

^a^n = 8

**Fig. 6:**
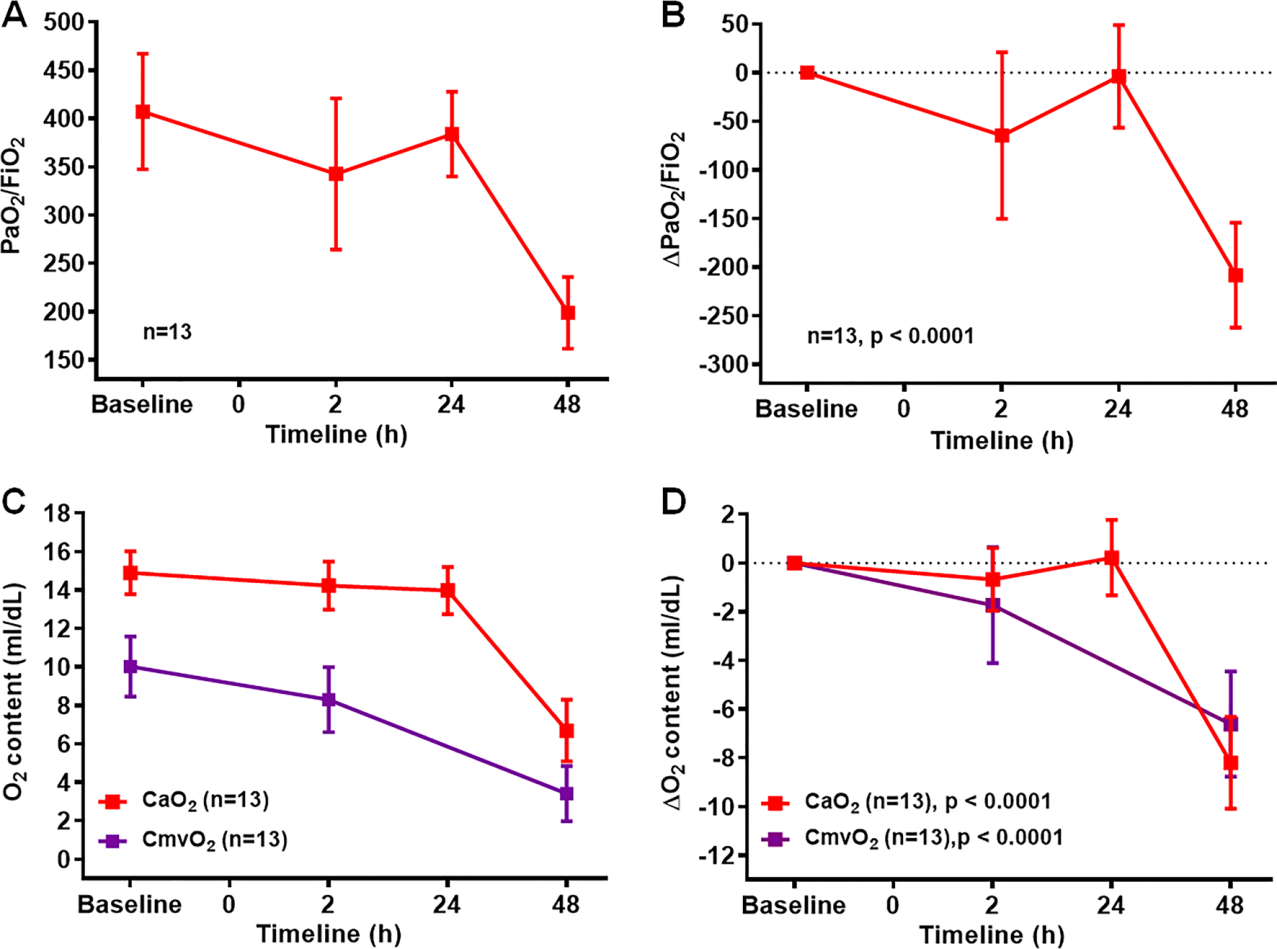
PaO_2_/FiO_2_ and total oxygen content reduced in smoke inhalation induced lung injury. **A** The ratio of PaO_2_ and FiO_2_, designated as “PaO_2_/FiO_2_” was measured in SI group of animals at baseline, 2 h, 24 h, and 48 h time points. **B** Delta PaO_2_/ FiO_2_ (ΔPaO_2_/FiO_2_) values were calculated between baseline and different time points. **C** Total arterial oxygen content (CaO_2_) and total mixed venous oxygen content (CmvO_2_) levels were measured in SI group at baseline, 2 h, 24 h and 48 h time points. **D** Delta CaO_2_ (ΔCaO_2_) and delta CmvO_2_ (ΔCmvO_2_) values were calculated between baseline and different time points (**D**). Delta value for each parameter represented the difference with the corresponding baseline values. PaO_2_/FiO_2_ and CaO_2_ and levels were measured at 24 h for n = 8 and n = 6 respectively; CmvO_2_ level was not measured at 24 h time line. Smoke inhalation started at 0 h time point. A p value of < 0.05 was considered statistically significant

Previous studies have documented PaO_2_/FiO_2_ ratio to assess the level of hypoxemia in the animal model [17, 24]. We also demonstrated significant reduction in PaO_2_/FiO_2_ ratio at 48 h post smoke exposure (198.87 ± 37.13), with 40% of animals having values less than 170 (Fig. 6A). The difference in the ΔPaO_2_/FiO_2_ value between the baseline and at 48 h post smoke exposure was approximately 208 (Fig. 6B, p < 0.0001). Furthermore, hematocrit (Hct), hemoglobin (Hb), and fractional oxyhemoglobin (FO_2_Hb) values were reduced 48 h after smoke inhalation compared to baseline (Table 2, p < 0.001 to = 0.02). As expected, total arterial oxygen content (CaO_2_) and mixed venous oxygen content (CmvO_2_) of blood were also significantly reduced 48 h after smoke inhalation compared to the corresponding baseline values (Figs. 6C, D, p < 0.0001). There were no significant changes in heart rate (HR), temperature (Temp), mean arterial pressure (MAP) and pulmonary arterial pressure (PAP) (Table 2).

### Effect of smoke inhalation on lung parenchyma

**Fig. 7:**
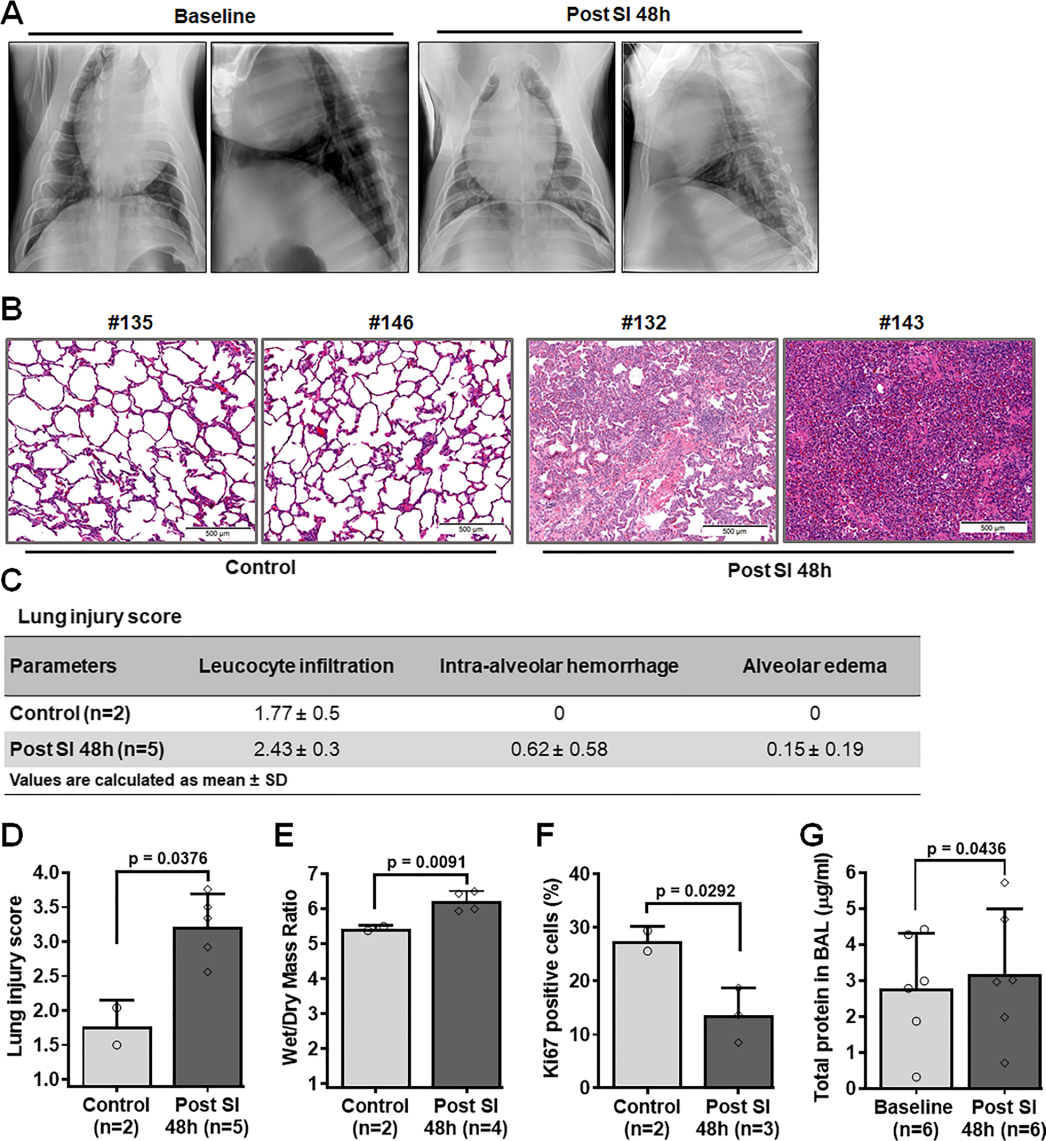
Effect of smoke inhalation in lung parenchyma. **A** Ventral-dorsal and lateral view chest x-rays at baseline and 48 h after smoke inhalation. Hematoxylin and eosin (H&E) staining on two sets of paraffin embedded lung tissue sections of control and SI animals (**B**). Statistical analysis of lung injury score between control and SI animals (**C**, **D**). **E** Statistical analysis of wet/dry weight (W/D) ratio between control and SI animals. **F** Percentage of Ki67 positive cells on the paraffin embedded lung tissue sections of control and SI animals. **G** Quantification of total protein concentration of BAL fluid samples in SI group at baseline and 48 h post smoke inhalation. A p value of < 0.05 was considered statistically significant

Smoke inhalation has been reported to increase capillary leakage [25, 26]. Consistent with previous studies, we observed diffuse, bilateral infiltrates on repeated radiographic assessment of lung injury with chest x-rays in both ventral-dorsal and lateral views at 48 h after smoke injury (Fig. 7A). In contrast, both lungs were normal in the chest x-rays taken before smoke inhalation (Fig. 7A). Histologic examination of lung tissue 48 h post smoke exposure showed an increase in the number of leukocyte infiltration, intra-alveolar hemorrhage, and alveolar edema compared to the control animal group (Figs. 7B, C); and the overall lung injury score was significantly increased (Fig. 7D; p = 0.0376). We also observed an increase in the average wet-dry weight (W/D) ratio of lung tissues 48 h after smoke exposure (6.233 ± 1.14) compared to the control animals (5.445 ± 0.36) (Fig. 7E, p = 0.0091). Ki67 immunohistochemistry was performed in paraffin embedded lung tissue sections of two control and two smoke inhalation animals. Lung tissue sections of animals at 48 h post smoke inhalation showed a statistically significant decrease in the number of proliferative cells compared to the control animals (Fig. 7F, p = 0.0292). Furthermore, BAL fluid samples taken from six animals showed significant increase in the total protein concentration of BAL fluid 48 h post smoke inhalation compared to the baseline (Fig. 7G, p = 0.0436).

### Effect of smoke inhalation on IL-6 expression level

**Fig. 8:**
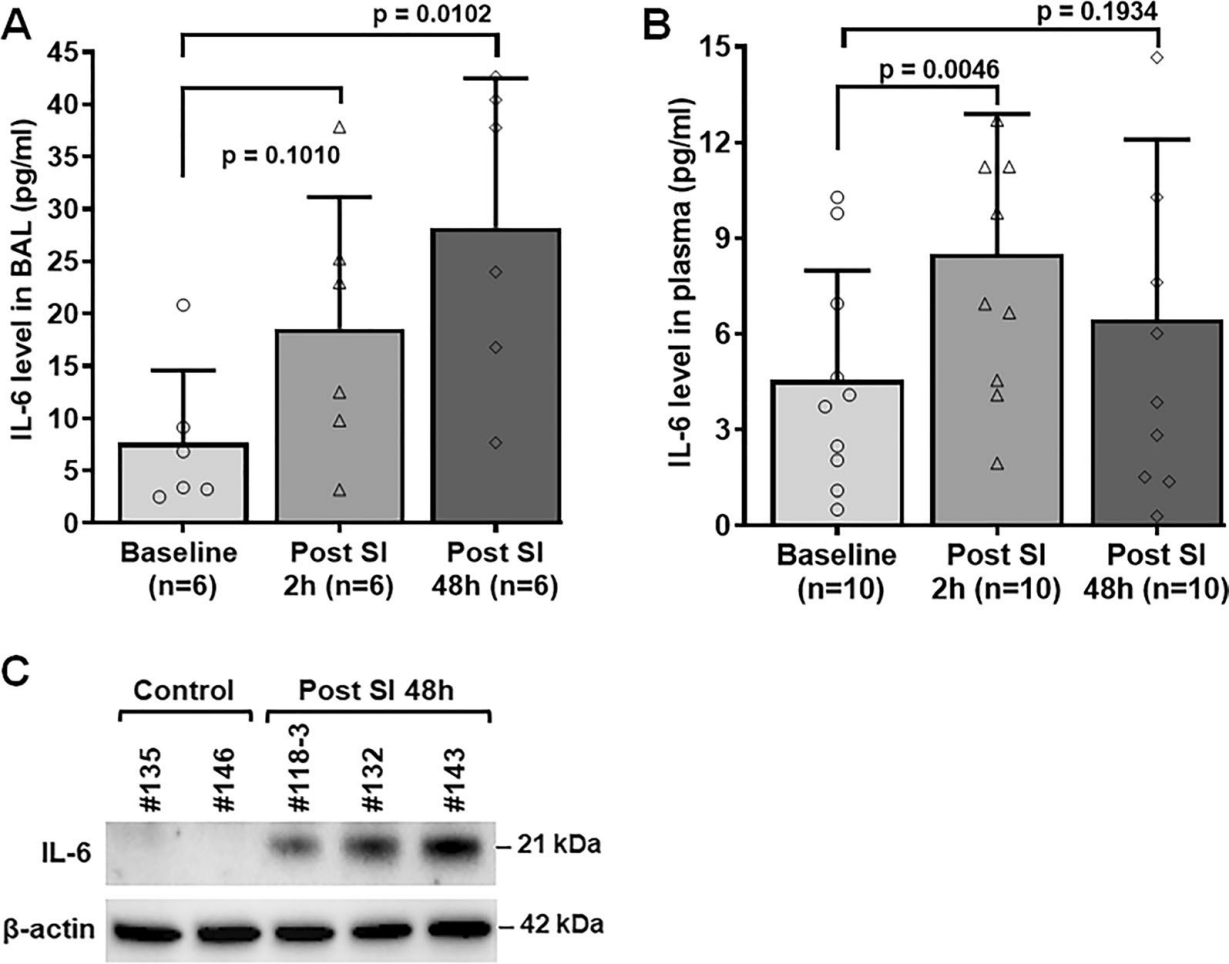
Effect of smoke inhalation on IL-6 expression. **A** IL-6 expression level in BAL fluid samples of SI animals at baseline, 2 h and 48 h time points. **B** IL-6 expression level in plasma samples of SI animals at baseline, 2 h and 48 h time points. **C** Immunoblot analysis of IL-6 expression level in fresh frozen lung tissues of SI and control animals. A p value of < 0.05 was considered statistically significant

We observed a significant increase in IL-6 level in BAL fluid samples at 48 h post smoke exposure (Fig. 8A, p = 0.0102), and a marginal increase at 2 h time point (Fig. 8A, p = 0.1010) compared to the baseline level. IL-6 immune assay analysis in plasma samples of 10 animals also demonstrated a significant increase in IL-6 level in SI animals at 2 h smoke inhalation compared to the baseline (Fig. 8B, p = 0.0046). However, no significant increase was observed in animals at 48 h post smoke inhalation (Fig. 8B, p = 0.1934). Increased IL-6 expression level obtained in BAL fluid and plasma sample immunoassay analyses were further validated by the robust upregulation of IL-6 expression level in immunoblotting of lung tissue lysates at 48 h post smoke inhalation in SI animals compared to the control animals (Fig. 8C).

## Discussion

ARDS arises from diverse insults in the lungs, and a variety of lung injury animal models have been developed to study the pathophysiological processes [3, 27]. Frequently used models for ALI includes intravenous infusion of oleic acid [28, 29], repeated bronchoalveolar lavage with saline [30, 31], and intravenous infusion of endotoxin [32, 33]. These models studied acute lung injury manifested within 24 h. In the current model, we wished to focus on the acute phase of injury after smoke inhalation; extrapolating from our experience with human smoke inhalation, the effects of smoke injury typically peak at 48–72 h post-exposure. We did not desire to confound the current study with sub-acute or chronic effects of ARDS. As such, ours is an acute model and does not reflect the long-term effects of ARDS, i.e. tissue remodeling and fibrosis.

Furthermore, no large animal models of ARDS from isolated smoke inhalation injury exists in the literature. Our main goal was to contribute to the scientific community by developing such a model that is reliable and reproducible. Smoke inhalation is a major predisposing factor for the development of ARDS and is considered together with burn injury in several animal models for smoke/burn injury-induced ARDS [17, 20–22]. However, there is no suitable large animal model available for isolated smoke inhalation-induced ARDS that replicates human ARDS without confounding variables such as cutaneous burn injury.

The present study utilized oak wood to generate smoke from the smoke generator. Oak wood is easily available and its composition and particle size has been well -characterized [34, 35]. Additionally, commercially-available oak are compatible with the smoke generator system used in the study. Oak wood is mainly composed of cellulose (41–46%), hemicellulose (19–22%) and lignin (29–30%), together with resins and variable amounts of water and inorganic matter [34]. Oak wood generates ~ 2 g fine particulate per kg of wood burned which composed primarily of organic carbon (~ 50%) and elemental carbon (~ 3%), and also include ions and elements [35]. Some of the organic compounds found in fine particle mass are levoglucosan and other sugar derivatives, substituted syringols, guaiacol and substituted guaiacol, substituted benzene and phenols, PAH and alkyl-PAH and phytosteroids [35]. We reported the development and validation of a large animal model for isolated smoke inhalation-induced ARDS. Following smoke exposure through an endotracheal tube, animal reproduced the entire set of clinical parameters for defining ARDS, such as significant decrease in SpO_2_ to approximately 65%, mean PaO_2_/FiO_2_ value of 196, diffuse bilateral pulmonary infiltrates on chest X-rays at 48 h post smoke inhalation, and no clinical evidence of cardiac failure or fluid overload as explained by ARDS Definition Task Force, 2012 [7]. We also documented significant decreases in PaO_2_ and the reciprocal increase in PaCO_2_. Studies have shown that increase in PaCO_2_ and ETCO_2_ levels correlate with the presence of physiological dead space in lungs and the increased in gradient between these two values corresponds with ARDS severity [36, 37]. We observed an increase in the gradient (PaCO_2_–ETCO_2_) from 7.67 at baseline to 14.21 at 48 h post smoke exposure. IL-6, one of the inflammatory cytokines involved in the development of ARDS started to rise 2 h time point in SI animals and sustained until 48 h in both plasma and BAL fluid samples. IL-6 expression level was also significantly upregulated in fresh frozen lung tissue samples of smoke exposed animals compared to control animals. We also observed a significant reduction of Ki67 positive cells in lung tissue of smoke exposed animals compared to control tissue indicating smoke exposure decreased normal lung parenchyma cell proliferation. In addition, evidence of significant lung tissue injury and inflammatory response to the injury were documented. These results indicated the deterioration of respiratory functions in the diseased lungs from smoke exposure.

Developing mimics of human ARDS in a large animal model such as pig has clear advantages, such as the ability to perform invasive procedures and serial blood analysis, and feasibility of documenting the respiratory function for an extended period of time. Moreover, monitoring the respiratory parameters in a mechanically ventilated animal allowed us to detail functional deterioration of the animal in a controlled environment after smoke inhalation. The detailed presentation of our model allows for others in the scientific community to independently confirm reproducibility, and hopefully use our model for investigations leading to clinical translation.

## Conclusion

We developed, for the first time, a novel large animal isolated smoke inhalation-induced ARDS model which mimics human ARDS and lacks confounding variables such as cutaneous burn injury or intra-abdominal sepsis. This model will help in better understanding the pathophysiological mechanisms involved in the process of smoke inhalation-induced ARDS and aid in the development of novel therapeutic strategies.

## Data Availability

Data are available upon reasonable request from the corresponding author.

## Abbreviations

ABG: Arterial blood gas
ALI: Acute lung injury
ANOVA: Analysis of variance
ARDS: Acute respiratory distress syndrome
BAL: Bronchoalveolar lavage
BCA: Bicinchoninic acid
CaO_2_: Arterial oxygen content
CmvO_2_: Mixed venous oxygen content
CA: Carotid artery
CO: Cardiac output
CVP: Central venous pressure
ELISA: Enzyme-linked immunosorbent assay
ETCO_2_: End-tidal carbon dioxide
FA: Femoral artery
FiO_2_: Fraction of inspired oxygen
HCO_3_: Hydrogen bicarbonate
HR: Heart rate
IL-6: Interleukin-6
MAP: Mean arterial pressure
PA: Pulmonary artery
PAP: Pulmonary artery pressure
PaCO_2_: Partial pressure of carbon dioxide
PaO_2_: Arterial oxygen partial pressure
PCWP: Pulmonary capillary wedge pressure
PEEP: Positive end-expiratory pressure
PIV: Peripheral intravenous catheter
PmvO_2_: Partial pressure of mixed venous oxygen
PVDF: Polyvinylidene fluoride
RR: Respiratory rate
SmvO_2_: Mixed venous oxygen saturation
SpO_2_: Peripheral oxygen saturation
TBST: Tris-Buffered Saline, 0.1% Tween® 20 Detergent
TV: Tidal volume
W/D: Wet-to-dry weight
